# A High-Throughput Method to Analyze the Interaction Proteins With p22 Protein of African Swine Fever Virus *In Vitro*

**DOI:** 10.3389/fvets.2021.719859

**Published:** 2021-09-06

**Authors:** Xuejiao Zhu, Baochao Fan, Junming Zhou, Dandan Wang, Huiying Fan, Bin Li

**Affiliations:** ^1^Institute of Veterinary Medicine, Jiangsu Academy of Agricultural Sciences, Key Laboratory of Veterinary Biological Engineering and Technology, Ministry of Agriculture, Nanjing, China; ^2^Jiangsu Co-innovation Center for Prevention and Control of Important Animal Infectious Diseases and Zoonoses, Yangzhou, China; ^3^Jiangsu Key Laboratory for Food Quality and Safety—State Key Laboratory Cultivation Base of Ministry of Science and Technology, Nanjing, China; ^4^School of Life Sciences, Jiangsu University, Zhenjiang, China; ^5^College of Veterinary Medicine, South China Agricultural University, Guangzhou, China; ^6^School of Food and Biological Engineering, Jiangsu University, Zhenjiang, China

**Keywords:** African swine fever virus, protein p22, GO KEGG pathways analysis, liquid chromatography, mass spectrometry

## Abstract

African swine fever virus (ASFV) has been identified as the agent of African swine fever, resulting in a mortality rate of nearly 100% in domestic pigs worldwide. Protein p22 encoded by gene KP177R has been reported to be localized at the inner envelope of the virus, while the function of p22 remains unclear. In this study, p22 interacting proteins of the host were identified by a high-throughput method and analyzed by Gene ontology terms and Kyoto Encyclopedia of Gene and Genomes (KEGG) pathways; numerous cellular proteins in 293-T that interacted with p22 protein were identified. These interacting proteins were related to the biological processes of binding, cell structure, signal transduction, cell adhesion, etc. At the same time, the interacted proteins participated in several KEGG pathways like ribosome, spliceosome, etc. The key proteins in the protein–protein interaction network were closely related to actin filament organization and movement, resulting in affecting the process of phagocytosis and endocytosis. A large number of proteins that interacted with p22 were identified, providing a large database, which should be very useful to elucidate the function of p22 in the near future, laying the foundation for elucidating the mechanism of ASFV.

## Introduction

African swine fever (ASF) is caused by African swine fever virus (ASFV), a linear, large, double-stranded DNA virus that is the only member of the Asfarviridae family (1). ASFV is an enveloped DNA virus with genome length of 170–193 kbp (1). The genome encoded 151–167 open reading frames. ASFV is an icosahedral symmetric virus that replicates in the cytoplasm of infected cells. Warthogs, Bush pigs, and soft ticks are natural hosts of the virus, which can persist to infect without any signs of disease (2). Once introduced into domestic pigs, ASFV is a highly pathogenic virus and could spread directly among pigs, resulting in nearly 100% mortality (3). Typical clinical symptoms include high fever, cyanosis, hemorrhagic lesions, anorexia, and ataxia (4). The lesion tissues display severe pathological vascular changes, such as renal ecchymosis, skin erythema, and diffuse hemorrhages in lymph nodes, kidneys, lungs, and urinary bladder; pulmonary edema; disseminated intravascular coagulation; and thrombocytopenia (5).

The disease has caused serious economic losses to the pig industry and has a severe impact on the world. Especially in 2018, the breakout of ASF in China spread quickly over the country, threatening the pig industry severely (6). Recent studies have shown that some viral proteins are involved in the adhesion and entry of ASFV. Some encoded structural proteins are involved in genome replication and virus infection (7). It is reported that 15 of the 26 virus-encoded proteins were detected in the virus proteome with predicted transmembrane domains (8). However, some detected proteins remain uncharacterized. Among the detected proteins on the membrane, protein p22 (pKP177R) has been predicted to be externally located in the virion (9). Some studies have reported that p22 was localized around the virus factories rather than at the cell surface (10, 11). In another study, it was tricky that protein p22 was weakly detected throughout the cytoplasm, including the virus factories, but could be detected at the periphery of assembling and mature icosahedral particles. Protein p22 was localized at the inner envelope (12). Other viral membrane proteins, like p17, pE183L, p12, and pE248R, were also at the cell surface but were localized at precursor viral membranes and intracellular icosahedral particles within the viral factories (13–16). Some structural proteins have been reported to be involved in virus entry, like p12, pE248R, and pE199L; some are required for the assembly process, like protein p17 and pE183L (17). These proteins are localized at the membrane of the virus, helping the entry or assembly process of the virus. However, the receptors of ASFV are still unclear. In recent study, p22 was proven not to be involved in virus replication or virulence in swine by KP177R gene deletion in recombinant virus (18). It might be due to the potential replacement of the KP177R gene by one of the L101L genes; there might be a potential overlapping in the function of these two genes. Therefore, the function of p22 is still unknown.

In this study, we studied the proteins that interacted with p22 of ASFV by proteomics analysis. Although a large number of structural protein studies have been performed, further researches on the function and molecular mechanism are desperately in need and will help prevent and control the spread of the disease.

## Materials and Methods

### Sample Preparation

Gene KP177R (p22) of the ASFV and tagged by hemagglutinin (HA) at C terminus was synthesized into the plasmid pcDNA-3.1(+) by the GeneScript Corporation (Shanghai, China) and sequenced correctly. The 293-T cells were grown into 80% confluency in Dulbecco's modified Eagle's medium, supplemented with 10% fetal bovine serum (FBS) and antibiotics (penicillin/streptomycin) (Thermo Fisher, MA, USA) in tissue culture plates. Cells were maintained at 37°C in a humidified atmosphere and supplemented with 5% CO_2_. The cells were then separately transfected with pcDNA3.1(+)-p22-HA and pcDNA3.1(+) (1 μg each) by Lipofectamine 3000 according to the instruction of the manufacturer (Thermo scientific, MA, USA) and were proven successfully expressed in 293-T cells. At 24 h post-transfection, p22-expressed or mock cells were washed once in cold phosphate-buffered saline (PBS) and suspended in 1 ml of cold immunoprecipitation (IP) buffer (Beyotime, Shanghai, China) (50 mM Tris-HCl, pH 7.4, 150 mM NaCl, 1 mM EDTA) supplemented with 0.5% Nonidet P-40 Substitute (NP-40, Fluka Analytical) on ice with 1% protease inhibitor cocktail (Roche, Shanghai, China). Cells were lysed for 30 min at 4°C with constant rotation, and the lysates were cleared by centrifugation at 5,000 × g for 5 min; lysate was removed for Western blot analysis (whole-cell lysate fraction). The remaining lysate was incubated with 1 μg of anti-HA antibody (Santa Cruz, Shanghai, China) overnight at 4°C, and then pre-coupled to 40 μl of A/G Plus agarose beads for 4 h at 4°C according to the instruction of the manufacturer. The immune complexes were precipitated, washed, and subjected to SDS-PAGE gels and Western blotting analysis.

### Liquid Chromatography Tandem Mass Spectrometry (LC-MS/MS) Analysis and Data Processing

**Figure 1:**
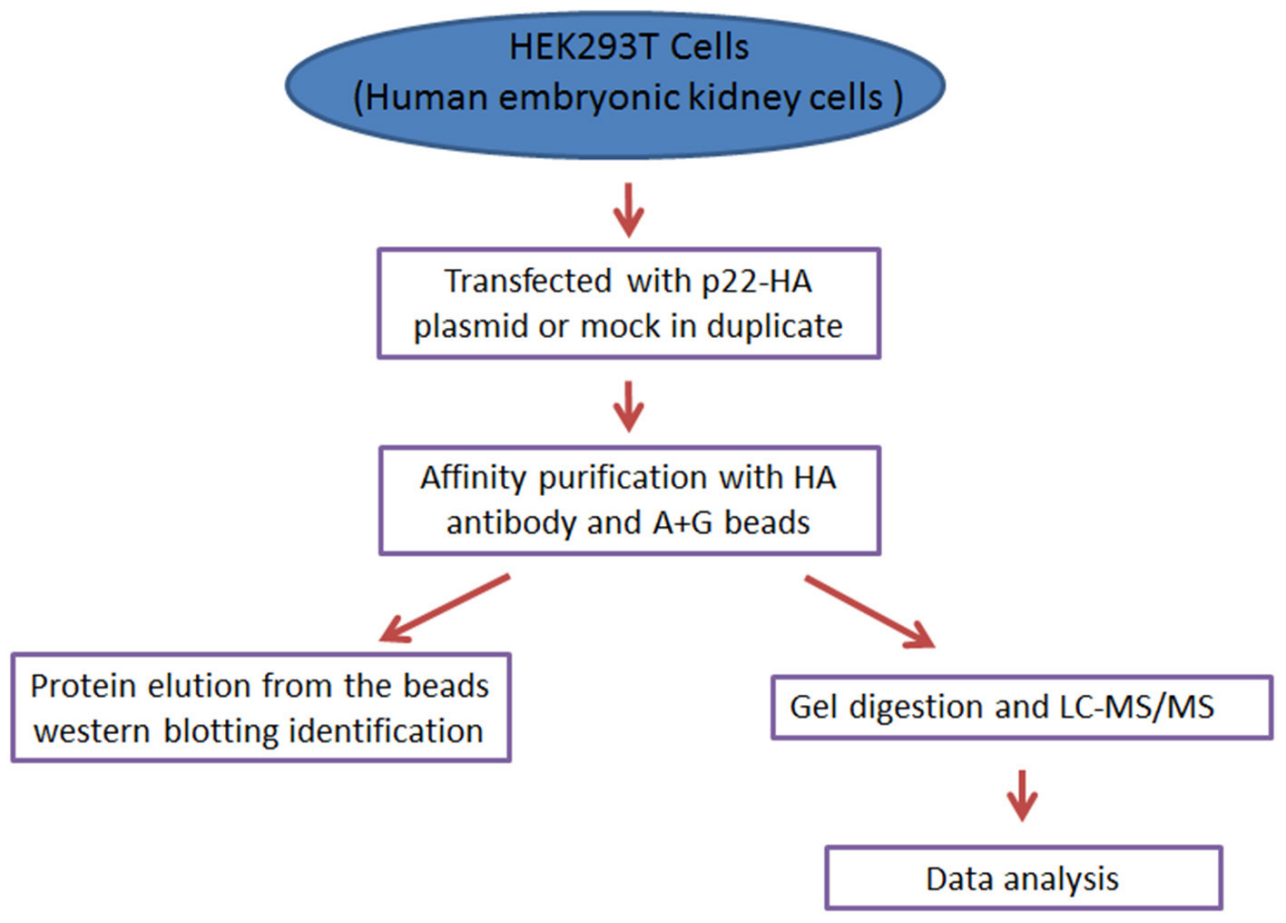
The concise procedure for sample preparation and process.

The preparation of peptides for MS of triplicate samples of each group and LC-MS/MS were all performed by the Shanghai Applied Protein Technology Company, and the LC-MS/MS was executed on an Q Exactive HF mass spectrometer (Thermo Scientific, MA, USA). In order to exclude possible contaminants, the databases were deleted in p22-immunoprecipitated proteins, which were obtained in mock immunoprecipitated proteins (the brief procedures are seen in Figure 1).

#### Western Blot

293-T cells were transfected with 1 μg of pcDNA3.1-p22-HA or mock plasmid for 24 h. At 24 h post-transfection, 293-T cells were lysed with lysis buffer containing 1% protein inhibitor. The cell lysates were subjected to SDS-PAGE and transferred onto 0.22-μm nitrocellulose membranes (Pall, Port Washington, NY, USA). Then, the membranes were incubated with 5% defatted milk at room temperature for 2 h, washed with PBS containing 0.05% Tween 20 three times, followed by anti-HA rabbit polyclonal antibody incubation at 4°C overnight, washed with PBST three times, and then incubated with horseradish peroxidase-conjugated goat anti-rabbit IgG secondary antibody at room temperature for 1 h. After washing three times with PBST, detection was performed using the ECL Kit (Thermo Fisher Scientific).

#### Indirect Immunofluorescence Assay (IFA)

293-T cells were transfected with 1 μg of pcDNA3.1(+)-p22-HA or mock plasmid for 24 h. At 24 h post-transfection, cells were fixed in 4% paraformaldehyde at 4°C for 30 min, and then the cell membranes were permeabilized with PBS containing 0.2% Triton X-100 for 5 min. The cells were incubated with 1:200 diluted anti-HA antibody at 37°C for 1 h. Then, the cells were incubated with Alexa Fluor 555-conjugated goat anti-rabbit IgG at 1:400 dilution at 37°C for 1 h and washed with PBS three times before examination.

### Gene Ontology (GO) Enrichment and Kyoto Encyclopedia of Gene and Genomes (KEGG) Pathway Analysis of p22 Interacting Proteins

GO is the concept of the combination of gene–gene functions and is designed to detect cell biological functions *via* a systematically dynamic and computational interpretation of genes, RNA, and proteins. It covers three main areas (19) including cellular components, molecular function, and biological processes. The KEGG database aims to systematically analyze genes and their related gene functions with an interacting network of molecules in the cells in a hierarchical order (20). GO enrichment and KEGG pathway analysis of p22 interacting proteins were conducted. DAVID (http://david.abcc.ncifcrf.gov/) used in this study is short for Database for Annotation, Visualization, and Integrated Discovery.

### Protein–Protein Interaction (PPI) Network Construction

The PPI plays an extremely important role in understanding cellular or systemic processes of cell growth, reproduction, and metabolism (21) and provides a platform for the annotation of functional, structural, and evolutionary properties of proteins. To further investigate the molecular mechanism of p22 of ASFV, PPI networks of p22 interacting proteins were constructed through the STRING database (http://www.string-db.org/). STRING is an online database that includes experimental as well as predicted interaction information and comprises >1,100 completely sequenced organisms. To select core genes from the PPI network, we analyzed the top biological structure of the network and obtained the proteins that directly interact with the target protein in the network. We selected the PPIs to construct the PPI network for visualization and analysis.

## Results

### Sample Identification

**Figure 2:**
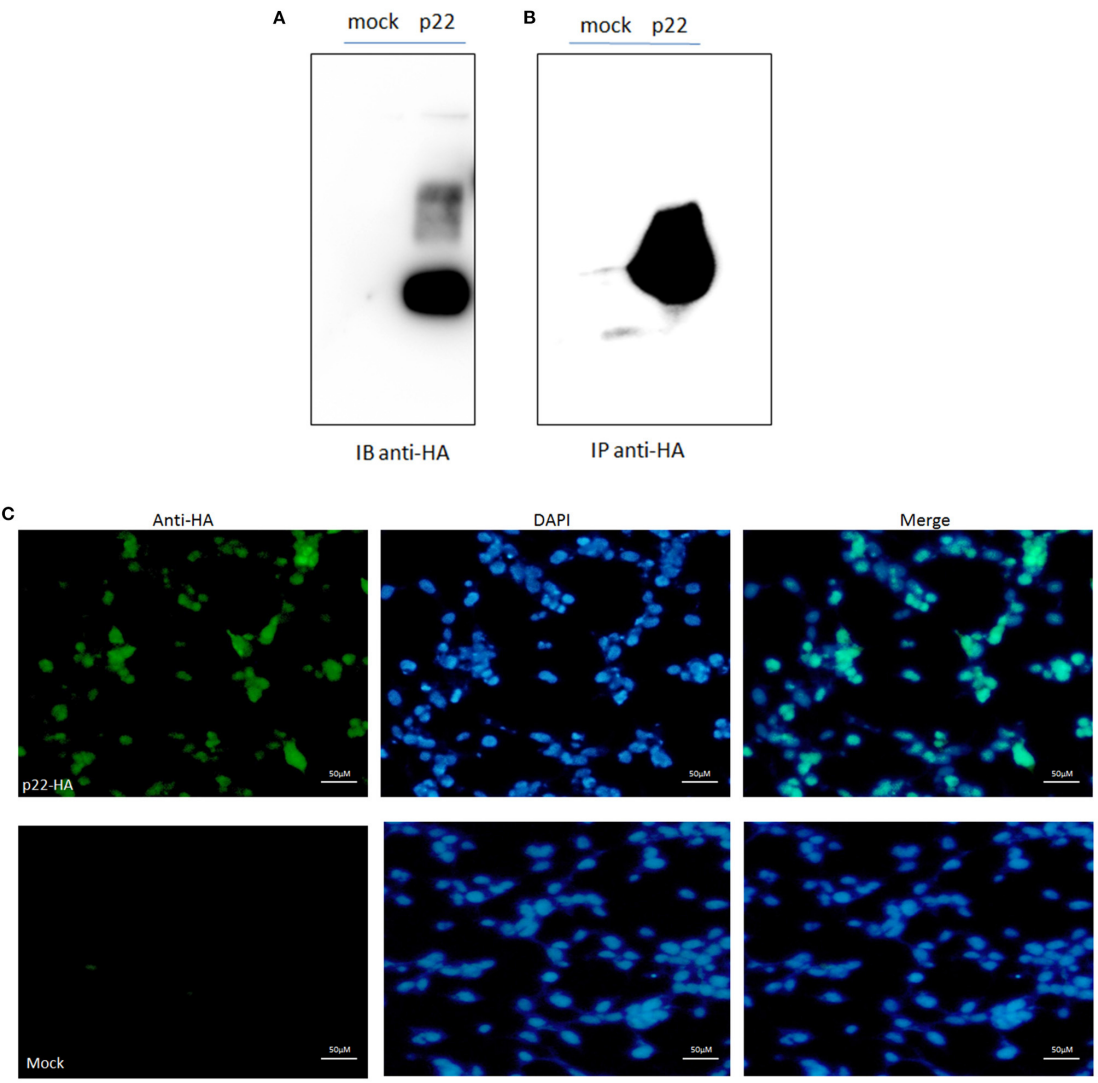
p22 expression identification. **(A)** pcDNA3.1(+)-p22-HA and its mock were transfected into the 293-T cells and at 24 h post-transfection. Cells were lysed and subjected to Western blotting analysis. The band of p22 of ASFV was shown as the predicted size. **(B)** The p22 protein was immunoprecipitated with HA and identified by Western blotting analysis. **(C)** IFA identification. The green fluorescence represented the p22 protein, and the DAPI stain represented the nucleus.

Protein p22 was expressed in the 293-T cells by Western blotting analysis; the band was shown as the predicted size of 25 kDa (Figure 2A). The sample of p22-HA and its mock immunoprecipitated for LC-MS/MS were also identified (Figure 2B). IFA analysis also proved the p22 protein expressed in 293-T cells in both the nucleus and plasma (Figure 2C).

### Enriched GO Terms Analysis

**Figure 3:**
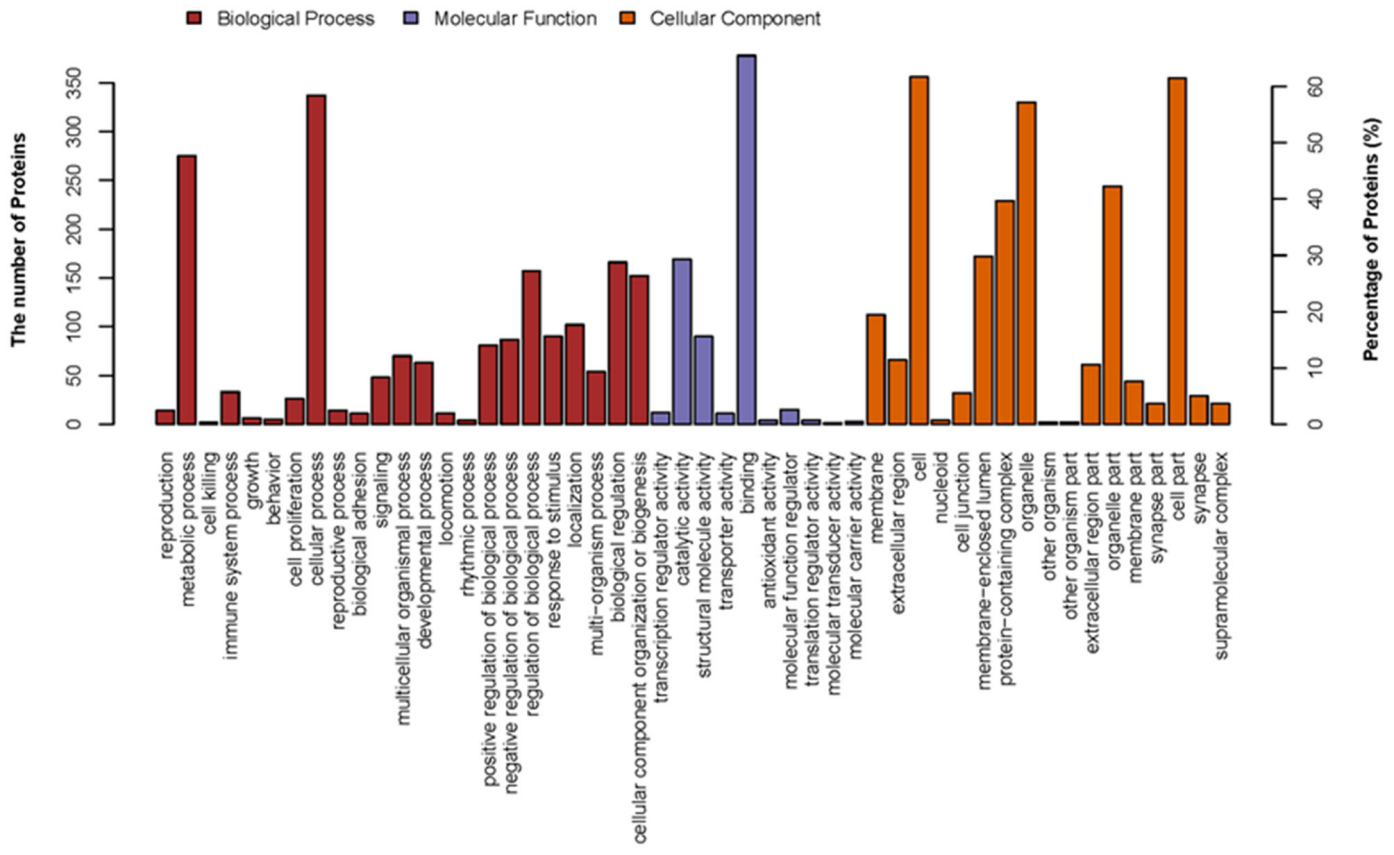
Distribution of GO terms on three groups: biological process, molecular function, and cellular component. Up coordinate represents the number of the proteins; right vertical coordinates represent the percentage of proteins.

In this study, to establish the host cell proteins or pathways that have been enriched in the p22 interacting partners' interaction networks, we performed gene ontology annotation and analysis of the target proteins in the p22 protein expressed 293-T cells to predict the biological function. There were 578 p22-interacted partners screened out compared with control samples in total (the data that repeated with mocked-interacted proteins were deleted to exclude the background contamination). From the GO map, thousands of enriched GO terms were obtained, and their corresponding proteins are shown in Supplementary Tables 1, 3. Go terms mainly covered three parts: biological process, molecular function and cellular component. A total of 359 proteins were related to the biological process (Figure 3). The top two enriched GO terms of the biological process were cellular process and metabolic process, followed by biological regulation, cellular component organization or biogenesis, etc. A total of 463 proteins were related to molecular function (Figure 3). Major enriched GO terms of molecular function were binding (as high as 378 proteins were included), catalytic activity, structural molecule activity, etc., indicating that p22 may play an important role in virus entry. For the cellular component, 374 proteins were involved. The GO terms analysis of the interacted proteins mainly included cell part, organelle, protein-containing complex, and membrane-enclose lumen membrane, indicating that p22 interacting partners may participate in cell structure maintenance. Collectively, the GO annotation and analysis of target proteins inferred that the p22 protein may participate in several processes such as protein binding, catalytic activity, and metabolism.

### KEGG Pathways Analysis

**Figure 4:**
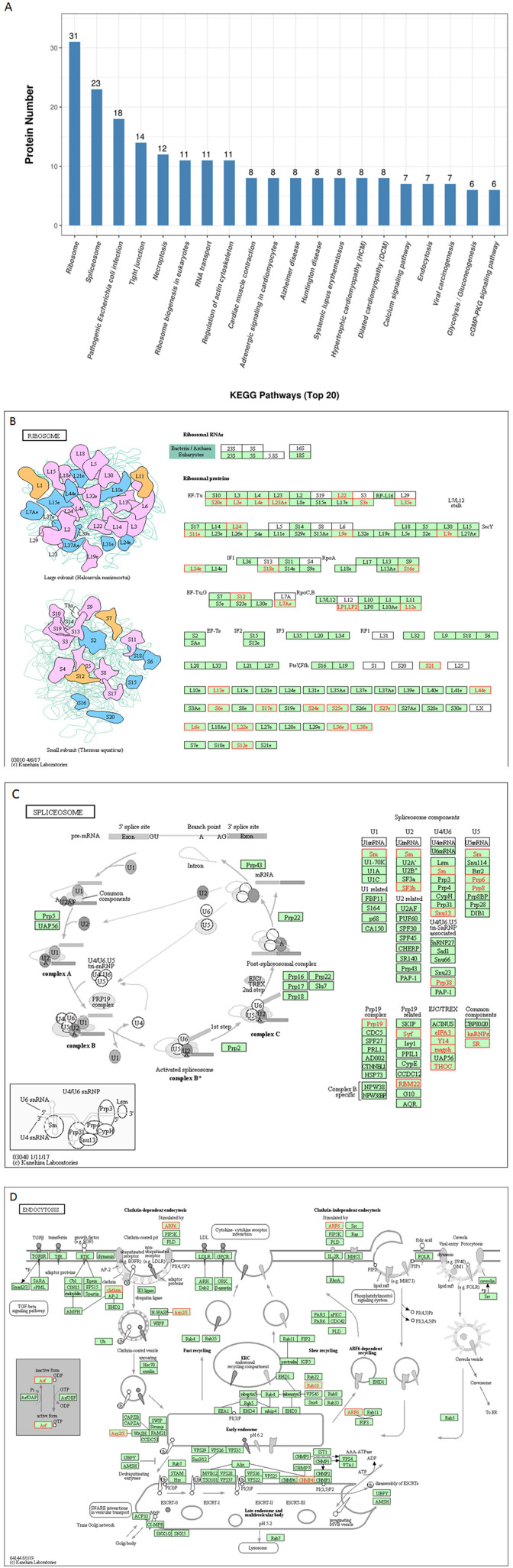
KEGG pathways analysis of p22 interacting proteins. **(A)** Top 20 enriched KEGG pathway distribution. KEGG pathways that p22 interacting proteins were involved in mainly were **(B)** ribosomes, **(C)** splicesome, and **(D)** endocytosis. The p22-interacting proteins in the KEGG pathways were marked in red.

To further predict the cellular pathways and signal transduction of p22-interacted host protein candidates, the KEGG and the top 20 enriched pathways with the highest representation of each term were enlisted (Figure 4A). A total of 165 KEGG pathways were screened out, and their corresponding protein numbers are shown in Supplementary Tables 2, 3. According to the result, the KEGG pathways in which the p22-related proteins were involved were ribosomes (Figure 4B) and spliceosome (Figure 4C), wherein the involved protein numbers were as high as 31 and 23, respectively. Furthermore, enrichment analysis also indicated that the proteins might participate in pathogenic *Escherichia coli* infection, tight junction, necroptosis, ribosome biogenesis in eukaryotes, RNA transport, regulation of actin cytoskeleton, cardiac muscle contraction, adrenergic signaling in cardiomyocytes, etc. It is noteworthy that KEGG pathway analysis showed that seven related proteins participated in endocytosis (Figure 4D), and six proteins were involved in cyclic GMP-dependent protein kinase (cGMP-PKG) signaling pathway and focal adhesion. Minor proteins (four proteins) participated in the cAMP signaling pathway and AMP-activated protein kinase (AMPK) signaling pathway. The KEGG enrichment analysis suggested that pathways involved in immune response, regulation of necroptosis, ribosome biogenesis, and endocytosis were preferentially targeted.

### PPI Network

**Figure 5:**
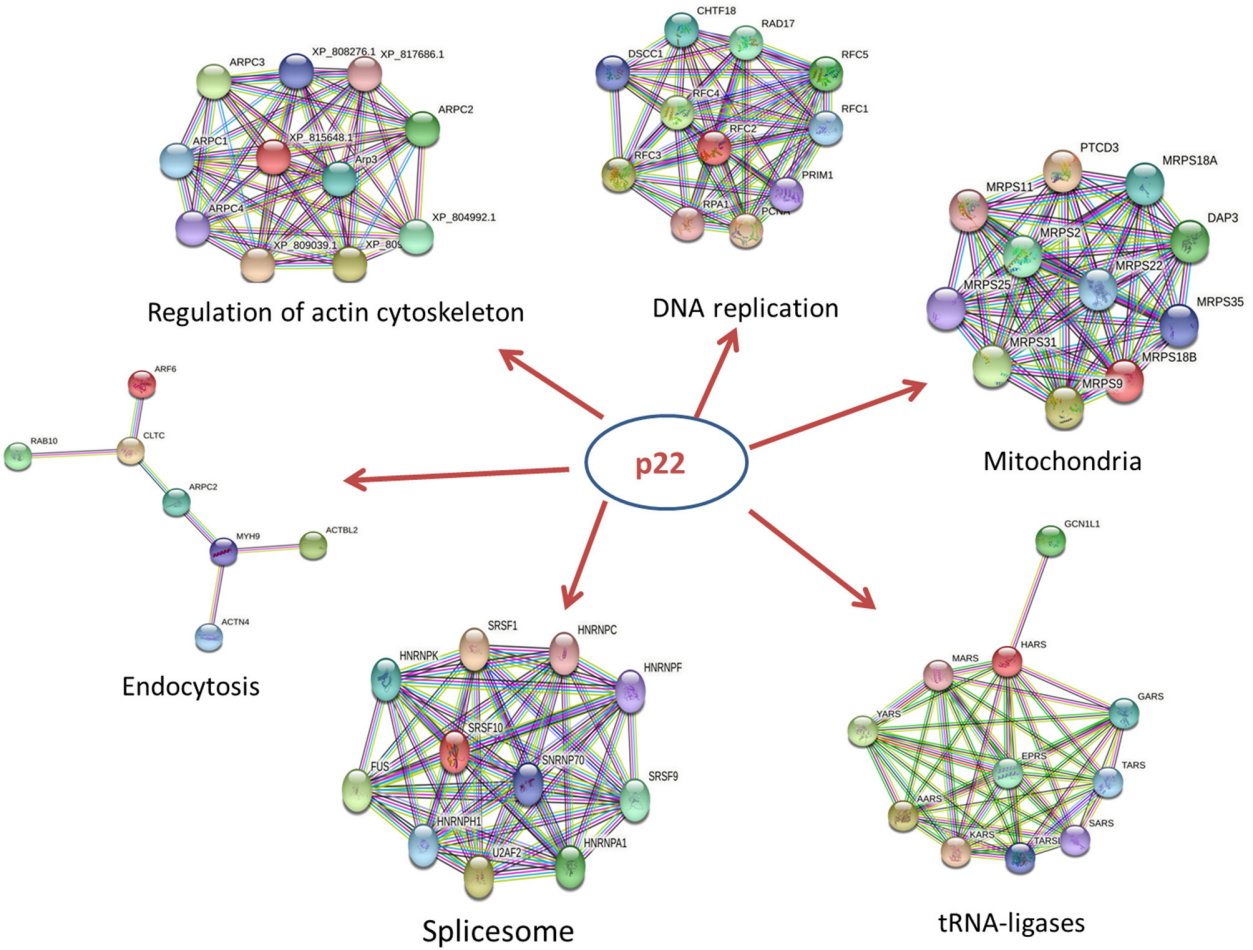
PPI network of key proteins that interacted with p22 in the endocytosis process. The size of each node in the PPI networks presents the connect degree of each gene. Those nodes that were not connected to any node were omitted in the network. The selected proteins that interacted with the p22 protein in the endocytosis process were connected as a network. The network was generated in the STRING database.

The p22-interacted proteins were placed in the STRING database for PPI analysis and visualization in Cytoscape software. The selected proteins that interacted with p22 protein in the endocytosis process were connected as a network. The proteins included myosin-9 (MYH9), actin-related protein 2/3 complex subunit 2 (ARPC2), actin-related protein 2, actin-related protein 2/3 complex subunit 1B, ADP-ribosylation factor 6 (ARF6), beta-actin-like protein 2 (ACTBL2), alpha-actinin-4 (ACTN4), clathrin heavy chain A (CLTC), and Ras-related protein-10 (RAB10). The PPI network contained eight nodes and 20 edges. All the hub proteins were at key positions in the interaction network. The nodes represented the interacted proteins, and the edges represented the interactions between these proteins. The selected proteins in the PPI network might relate to p22 more closely in the process of endocytosis. In addition to endocytosis, other PPI networks were also involved, including regulation of actin cytoskeleton, DNA replication, spliceosome, tRNA ligases, and mitochondria, implying that p22-interacted proteins functioned widely (Figure 5).

## Discussion

As the protein localized at the inner envelope of ASFV, the function of p22 is rarely known. In an attempt to acquire the p22 function, p22-interacted proteins of the host were identified by a high-throughput method and analyzed by GO terms and KEGG pathways; numerous cellular proteins in 293-T that interacted with p22 protein were identified. Although the facility to transfect 293-T cells made us select this cell type as the target cell, the interacted partners with p22 protein in the host cell derived from pig will be further investigated in the future. This study provides a large database and a useful tool to figure out the function of p22.

In this study, GO terms mainly covered three parts: biological process, molecular function, and cellular component. The top two enriched GO terms of the biological process were cellular process and metabolic process, implying that p22 might utilize the host proteins directly or indirectly to affect cell growth, function, and stability. Main enriched GO terms of molecular function were binding, catalytic activity. GO analysis revealed that the most significant ontology categories of molecular function of p22 interacting proteins is binding, suggesting a role of p22 as the protein at the inner envelop in virus binding and entry into the cell. The interesting result would inspire us to dig out the real function of p22 in virus entry.

The GO term analysis of the p22 interacting proteins in the cell component mainly included cell part, organelle, protein-containing complex, membrane-enclose lumen, and membrane; the results further verified the conclusion that p22 located at the membrane of the viron might participate in virus structure maintenance and contact with the host membrane *via* the binding and endocytosis process. Of course the suspicion needs further to be proven.

For KEGG pathways analysis, a large number of KEGG pathways were screened out (as high as 165); the KEGG pathways that p22 interacting proteins participated in mainly were ribosomes and spliceosome. Ribosomes are essential nanomachines for protein production and protein synthesis. The initial steps of ribosome biogenesis take place in the cell compartment. Spliceosome executes eukaryotic precursor messenger RNA (pre-mRNA) splicing to remove non-coding introns. It depends on the interactions of RNA–RNA, RNA–protein, and protein–protein. It is composed of several nucleoproteins and has the function of recognizing 5′ splicing site, 3′ splicing site, and branching point of mRNA precursor. It indicated that p22 interacting proteins mainly participated in the process of gene expression in the host cells, gave us a hint that p22 affected the gene and protein expression of cell host, and directly or indirectly affected the function of the biological process.

Furthermore, it is interesting that p22 interacting proteins were involved in pathogenic *E. coli* infection, tight junction, necroptosis, ribosome biogenesis in eukaryotes, RNA transport, regulation of actin cytoskeleton, cardiac muscle contraction, adrenergic signaling in cardiomyocytes, etc. The widely affected pathways reflected the wide range functions of p22 or its related proteins.

It is noteworthy that KEGG pathway analysis showed that seven of p22 interacting proteins participated in endocytosis. The results of the GO analysis indicated that a large number of p22 interacting proteins participated in binding. Above all, p22 was predicted to be involved in the entry process at the envelop of the virus.

At last, it was possible that other pathways had an important influence on the progression of ASFV entry *via* some biological process, such as cGMP-PKG signaling pathway, cAMP signaling pathway, and AMPK signaling pathway, which were screened out by KEGG analysis. cGMP is the intracellular second messenger that mediates the action of nitric oxide (NO) and natriuretic peptides (NPs), affecting a wide range of physiologic processes (22). cGMP/PKG signaling pathway was associated with the replication of some viruses (23, 24). cAMP is also one of the most common and universal second messengers; cAMP regulates pivotal physiologic processes including metabolism, secretion, calcium homeostasis, muscle contraction, cell fate, and gene transcription (25). AMPK is a central regulator of cellular energy homeostasis, regulating growth and reprogramming metabolism, as well as in cellular processes including autophagy and cell polarity (26). cAMP and AMPK are also closely connected with virus replication (27, 28). These involved pathways put forward the possibility that p22 and its interacting proteins might affect the replication of ASFV.

In those hub proteins connected in the PPI network, the ADP-ribosylation factor (Arf) protein family is part of the large Ras superfamily that encompasses small GTPases (29). Among this family, ARF6 stimulates actin polymerization, drives phagocytosis through multiple mechanisms, and assists autophagy as well (30). Other than Arf6, RAB10 also influences the GTPase activity (29), Rab10 is located on both Golgi and early endosomal/recycling compartments and plays an important role in lysosome exocytosis and plasma membrane repair (31). Alpha actinin belongs to the spectrin gene superfamily, which represents a diverse group of cytoskeletal proteins. Alpha actinin is an actin-binding protein. In non-muscle cells, it is involved in actin binding to the membrane. In skeletal, cardiac, and smooth muscle isoforms, it is localized to the Z-disc and analogous dense bodies and participates in anchoring the myofibrillar actin filaments. ACTN4 encodes a non-muscle, alpha actinin isoform, which is concentrated in the cytoplasm and involved in metastatic processes (32). MYH9 is involved in several important functions, including cytokinesis, cell motility, and maintenance of cell shape (33). ARPC2, actin-related protein 2/3 complex subunit 2, contains seven subunits, of which Arp2 and Arp3 belong to actin-related proteins (34). The activation of Arp2/3 complex could promote the synthesis of F-actin in the suitable condition (35). The Arp2/3 complex is involved in the rearrangement of the macrophage cytoskeleton and affected the phagocytosis of macrophages (36). Knockout of the Arp2/3 complex APC2 gene in mouse macrophages results in a decrease in F-actin polymerization and subsequent reduction in phagocytic capacity (37). In summary, the key proteins mentioned above and other hub proteins in PPI network were closely related to actin filament organization and movement, resulting in affecting the process of phagocytosis and endocytosis. Additional studies on the role of p22 in the process of endocytosis should be conducted. In addition to endocytosis, other PPI networks including regulation of actin cytoskeleton, DNA replication, spliceosome, tRNA ligases, and mitochondria were screened out, indicating that p22 interacting proteins functioned widely and participated in several biological processes.

## Conclusions

Although several studies have been reported to elucidate the pathogenesis of ASFV, the viral protein function remains unclear. In this research, the proteins in the host cells interacted with p22, and the signaling pathways they might participate in were screened out by a high-throughput method, laying the foundation to elucidate the function of p22. For the pig industry, it would also be advantageous to study the pathogenesis of the disease and to monitor and predict the outcome to control the disease in the near future.

## Data Availability Statement

The original contributions presented in the study are included in the article/Supplementary Material, further inquiries can be directed to the corresponding author/s.

## Author Contributions

BF: conceptualization. BF and JZ: methodology and resources. DW: investigation. XZ: writing and original draft. XZ and BL: writing. BL and HF: funding acquisition and supervision. All authors contributed to the article and approved the submitted version.

## Conflict of Interest

The authors declare that the research was conducted in the absence of any commercial or financial relationships that could be construed as a potential conflict of interest.

## Publisher's Note

All claims expressed in this article are solely those of the authors and do not necessarily represent those of their affiliated organizations, or those of the publisher, the editors and the reviewers. Any product that may be evaluated in this article, or claim that may be made by its manufacturer, is not guaranteed or endorsed by the publisher.

## Supplementary Material

Supplementary Table 1 GO terms and corresponding proteins.

Supplementary Table 2 KEGG pathways and corresponding proteins.

Supplementary Table 3 Proteins accession numbers and description.

## Abbreviations

ASFV: African Swine Fever Virus
LCMS: Liquid Chromatography Mass Spectrometry
GO: Gene ontology
KEGG: Kyoto Encyclopedia of Gene and Genomes
PPI: Protein-protein interaction
MYH9: Myosin-9
ARPC2: Actin-related protein 2/3 complex subunit 2
ARF6: ADP-ribosylation factor 6
ACTBL2: Beta-actin-like protein 2
ACTN4: Alpha-actinin-4
CLTC: Clathrin heavy chain A
RAB10: Ras-related protein-10
pre-mRNA: precursor messenger RNA
cGMP: Cyclic GMP
PKG: cGMP-dependent protein kinase
NO: nitric oxide
AMPK: AMP-activated protein kinase.
